# Risk Threshold for Starting Low Dose Aspirin in Pregnancy to Prevent Preeclampsia: An Opportunity at a Low Cost

**DOI:** 10.1371/journal.pone.0116296

**Published:** 2015-03-19

**Authors:** Emily Bartsch, Alison L. Park, John C. Kingdom, Joel G. Ray

**Affiliations:** 1 Western University, London, Ontario, Canada; 2 Institute for Clinical Evaluative Sciences, Toronto, Ontario, Canada; 3 Department of Obstetrics and Gynaecology, University of Toronto, Toronto, Ontario, Canada; 4 Department of Medicine, St. Michael's Hospital, University of Toronto, Toronto, Ontario, Canada; Medical Faculty, Otto-von-Guericke University Magdeburg, Medical Faculty, GERMANY

## Abstract

**Background:**

Preeclampsia (PE) increases maternal and perinatal morbidity and mortality. Based on a multitude of data from randomized clinical trials, clinical practice guidelines endorse using ASA to prevent PE in women who are “at risk.” However, data are lacking about the level of absolute risk to warrant starting ASA prophylaxis.

**Methods and Findings:**

We present two approaches for objectively determining the minimum absolute risk for PE at which ASA prophylaxis is justified. The first is a new approach—the minimum control event rate (CER_min_). The second approach uses a pre-existing concept—the minimum event rate for treatment (MERT). Here we show how the CER_min_ is derived, and then use the CER_min_ and the MERT to guide us to a reasonable risk threshold for starting a woman on ASA prophylaxis against PE based on clinical risk assessment. We suggest that eligible women need not be at “high risk” for preeclampsia to warrant ASA, but rather at some modestly elevated absolute risk of 6–10%.

**Conclusions:**

Given its very low cost, its widespread availability, ease of administration and its safety profile, ASA is a highly attractive agent for the prevention of maternal and perinatal morbidity worldwide.

## Background

Preeclampsia (PE), defined as new-onset hypertension after 20 weeks gestation often with significant proteinuria [1, 2] affects between 2.5% and 7.6% of pregnancies in the general population Worldwide [3]. PE is associated with increased maternal and perinatal morbidity and mortality [4], cited as the leading cause of admission to the intensive care unit during the puerperal period [5, 6], and is the fourth leading cause of maternal death [7]. Additionally, women with PE have a higher risk of placental abruption, and their fetuses have a higher risk of stillbirth, preterm delivery, and intrauterine growth restriction [8–10].

Acetylsalicylic acid (ASA) effectively reduces the risk of PE and its aforementioned sequelae [11–14], without producing adverse effects on the mother or fetus [3, 15]. Meta-analyses of randomized controlled trials (RCTs) demonstrate a 17% relative risk reduction (RRR) for PE with ASA over placebo [11]. ASA prophylaxis started early in the pregnancy, at 12–16 weeks, confers a 53% RRR for PE and a 91% RRR for severe PE [12–14]. Important benefits are also seen for the fetus, such as a reduced risk of preterm birth, intrauterine growth restriction and neonatal death and morbidity [12–14] (S1 Table).

Current guidelines for obstetricians and midwives endorse using ASA for the prevention of PE in women at risk of this condition [1]. However, guidance about what level of PE risk warrants ASA prophylaxis is lacking. This uncertainty has resulted in inconsistencies in recommendations and possible underuse of ASA [16]. Without an agreed upon threshold of risk for PE, based upon valid algorithms, it is problematic for clinicians to identify which women should receive ASA.

Establishing a threshold level of risk for PE is needed by clinicians, since there is a growing body of evidence that details how different clinical risk factors can forecast PE risk. Based on this evidence, estimates of PE risk range from less than 4% for women with no risk factors up to around 25% for women with just one risk factor, such as chronic hypertension [17, 18]. Other clinical risk factors also heighten the risk. For example, the risk of developing PE is 10% in obese women with a body mass index of 30 kg/m^2^ or greater, increasing to 16% if her systolic blood pressure is also over 120 mm Hg [18].

We present two approaches for objectively determining the minimum risk for PE at which ASA prophylaxis is justified. The first is a new approach—the *minimum control event rate* (*CER*_*min*_). The second approach uses a pre-existing concept—the *minimum event rate for treatment (MERT)* [19]. Here we show how the CER_min_ is derived, and then use the CER_min_ and the MERT to guide us to a reasonable risk threshold for starting a woman on ASA prophylaxis against PE based on clinical risk assessment.

## Methods and Results

### Threshold Number Needed to Treat (NNT_T_)

A useful measure of ASA efficacy in this context is the *number needed to treat* (*NNT*)—the number of patients a clinician needs to treat in order to prevent one target outcome, such as a stroke [19]. The *NNT* has been widely adopted to communicate treatment effect sizes in a clinically meaningful way [20]. In 2001, Sinclair et al. introduced the idea of using a threshold for the *NNT*—the *NNT*_*T*_—which is the maximum number of patients a clinician would be willing to treat to prevent one target outcome [21]. The *NNT*_*T*,_ which aims to guide treatment recommendations, is determined by estimating the clinical value of preventing one target event and identifying the number of patients that would need to be treated such that this benefit is equivalent to the negative consequences of treating the same number of patients—both in terms of adverse events and costs [21]. In the specific case of using ASA to prevent PE and related outcomes, we herein use the *number needed to prevent* (*NNP*) and the threshold *NNP*_*T*_.

The value of preventing one target event can be estimated in quality adjusted life years (QALY) gained. To solve for the *NNP*_*T*_, we assign a $50 000 value per QALY gained, in accordance with the conventional willingness to pay threshold accepted in cost-effectiveness analysis research [22, 23].

### Deriving the Minimum Control Event Rate (CER_min_)

The CER_min_ is the minimum disease event rate in the placebo control group(s) of one or more RCTs in which the intervention (e.g., ASA) is cost-effective.

To derive the CER_min_ for ASA prophylaxis, we consider both the NNP_T_ and the known prevalence of PE in the ASA treatment groups within the RCTs—the *treatment event rate* (*TER*).

First, we isolate the CER, in terms of TER and NNP:

$$\begin{array}{l} ARR=CER-TER,\;\text{where ARR is the absolute risk reduction}.\\ \text{Also},ARR=1/NNP\\ \text{and}\;1/NNP=CER-TER\\ \text{such that}\;CER=TER+1/NNP \end{array}$$ (Equation 1)

In the case of CER_min_, we focus on the NNP_T_, which was derived using the equation by Shimbo et al. [24], a simplification of that by Sinclair et al. [21]:

$$NNP_{T}=QALYsgained\times \$50 000/DC$$

where DC is the direct cost of the treatment for one patient, and QALYs gained is the number of quality adjusted life years gained by avoiding one target event.

Now, substituting the equation for NNP_T_ provided by Shimbo et al. [24] into Equation 1, we can approximate the CER_min_ as follows:

$$\begin{array}{l} CER_{min}=TER+1/\left[\left(QALYsgained\times \$50 000\right)/DC\right]\\ =TER+\left[DC/\left(QALYsgained\times \$50 000\right)\right] \end{array}$$

Hence, we arrive at the final equation:

$$CER_{min}=TER+\left[DC/\left(QALYsgained\times \$50 000\right)\right]$$ (Equation 2)

### Application of the CER_min_ to preeclampsia prophylaxis with ASA

Using Equation 2 for the CER_min_ we fill in the known values for ASA prophylaxis, namely, the DC, QALYs gained, and TER.

The DC of taking 81 mg baby ASA daily for one year varies between $14 to $24 [25, 26], and ASA can be purchased at any drug store. Assuming that a woman needs to take ASA prophylaxis from 12 to 38 weeks gestation, she will pay a DC of $10.

**Fig 1:**
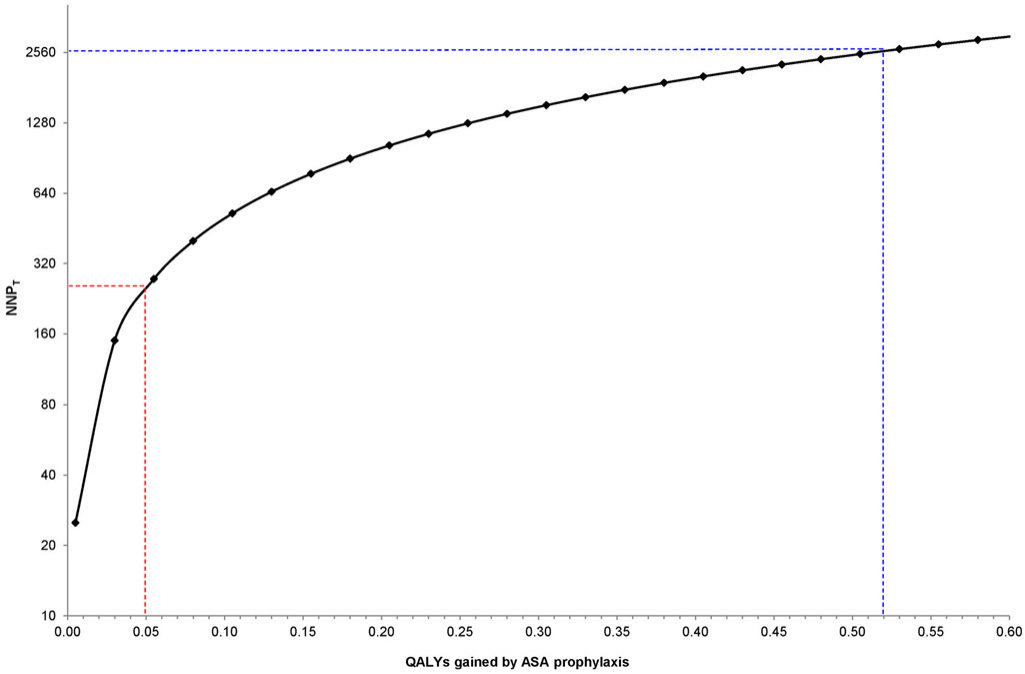
Threshold number needed to prevent (NNP_T_) at varying quality-adjusted life years (QALYs) gained per preeclampsia event avoided. NNP_T_ calculated as: (QALYs gained x $50000) / DC [24], assuming a direct cost (DC) of ASA of $10 per pregnancy. The dashed blue lines show that, at 0.52 QALYs gained by ASA prophylaxis [1], a clinician would be willing to give ASA prophylaxis to as many as 2600 women—the NNP_T_. For a more modest amount of 0.05 QALYs gained, the NNP_T_ would be 250 (dashed red lines). The NNP_T_ is presented on a log_2_ scale for ease of viewing.

Next, we consider QALYs gained per case of preeclampsia prevented. In the case of ASA prophylaxis, one analysis considered the benefits to both the mother and baby, arriving at a value of 0.52 QALYs gained per pregnancy [1]. Nonetheless, a value of 0.52 QALYs gained per pregnancy may be a generous estimate, so we determined NNP_T_ values at various QALYs gained, starting at as low as 0.005 QALYs gained (Fig. 1).

**Fig 2:**
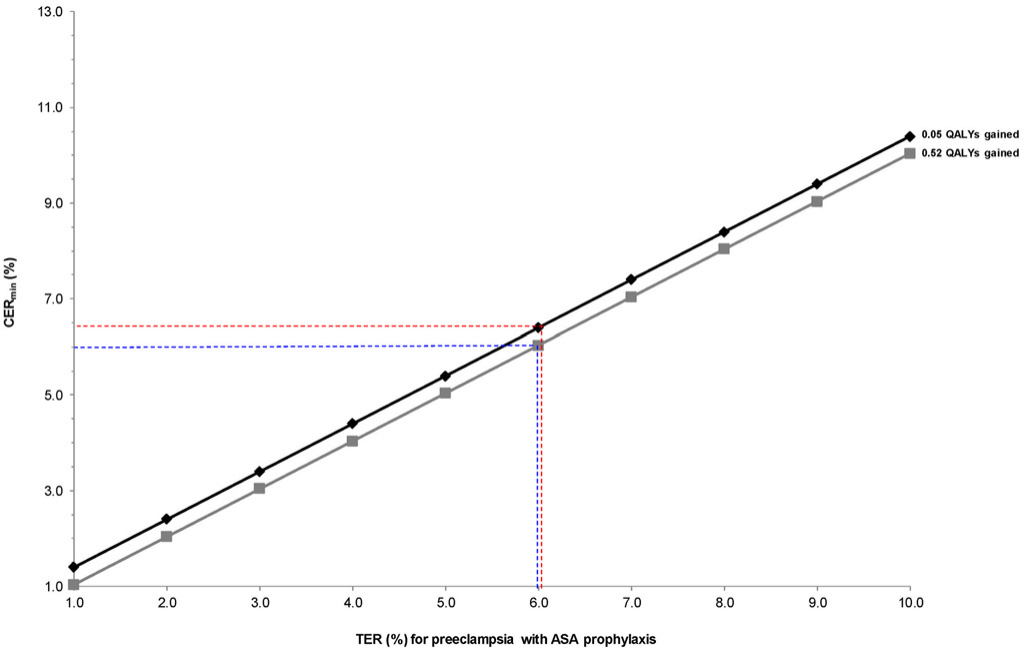
Minimum control event rate (CER_min_) for preeclampsia with varying treatment event rates (TER) for preeclampsia, at fixed values of 0.05 (black) and 0.52 (grey) quality-adjusted life years (QALYs) gained with ASA prophylaxis in pregnancy [1]. CER_min_ calculated as: TER + [DC / (QALYs gained x $50 000)], assuming a direct cost (DC) of ASA of $10 per pregnancy. The dashed blue lines indicate that at a TER for preeclampsia of 6.0% and a QALY gain of 0.52 [1], the CER_min_ for preeclampsia is close to the TER (6.0%). At a smaller QALY gain of 0.05, a TER of 6.0% corresponds to a slightly higher CER_min_ of 6.4% (dashed red lines).

In calculating the CER_min_ by Equation 2, we first fix the value for QALYs gained at 0.05 and 0.52, and observe how the CER_min_ changes with increasing TER (Fig. 2). We see that at a DC of $10, the TER very closely approximates the CER_min_ when QALYs gained is 0.52. At a QALY gain of 0.05, the CER_min_ is 0.4% higher than the TER.

**Fig 3:**
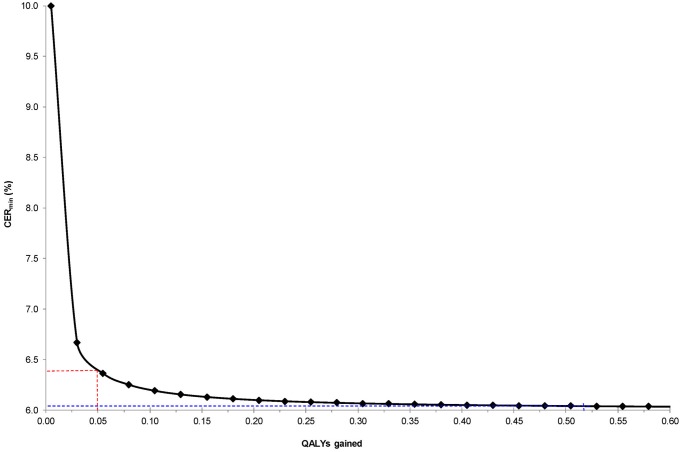
Minimum control event rate (CER_min_) for preeclampsia with varying quality-adjusted life years (QALYs) gained, at a fixed treatment event rate (TER) for preeclampsia of 6.0%. CER_min_ calculated as: TER + [DC / (QALYs gained x $50 000)], assuming a direct cost (DC) of ASA of $10 per pregnancy. The dashed blue lines indicate that at 0.52 QALYs gained [1], the CER_min_ for preeclampsia is close to the TER (6.0%). At a more modest gain of 0.05 QALYs, the CER_min_ increases slightly to 6.4% (dashed red lines).

If we fix the TER at 6.0%—a rate observed in many ASA RCTs [11, 12, 14]—we see how the CER_min_ changes with varying QALYs gained (Fig. 3). The CER_min_ declines with increasing QALYs gained, but these changes become very minor above a QALY gain of 0.05.

**Fig 4:**
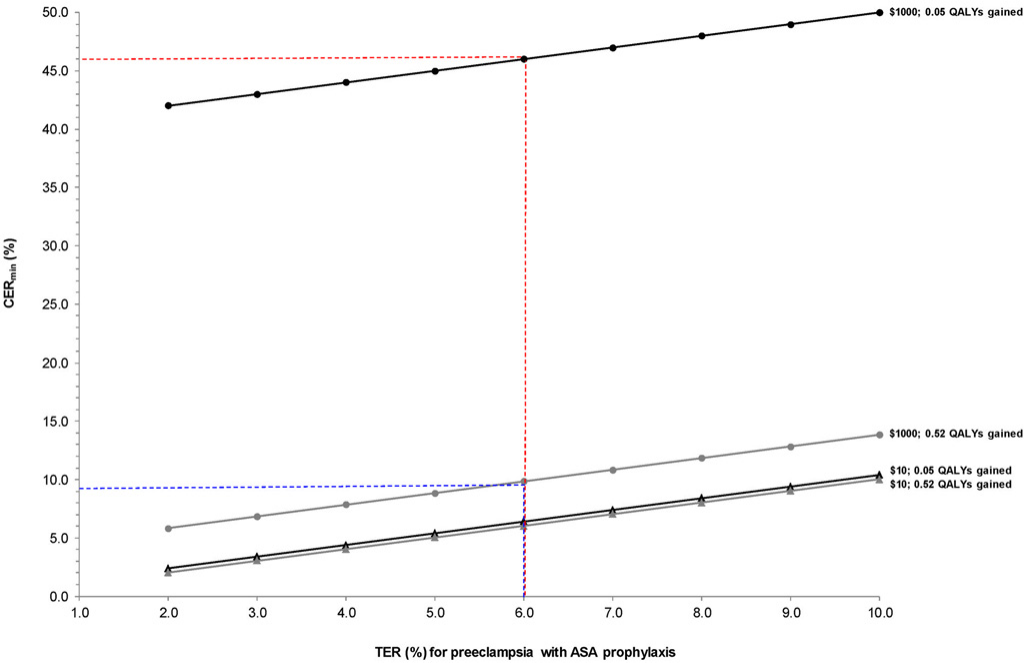
Minimum control event rate (CER_min_) for preeclampsia with varying treatment event rates (TER) for preeclampsia at fixed values of 0.05 (black) and 0.52 (grey) quality-adjusted life years (QALYs) gained with ASA prophylaxis in pregnancy [1], and varied by two direct costs (DC) of ASA: $10 (actual cost, triangles) and $1 000 (circles). CER_min_ calculated as: **TER + [DC / (QALYs gained x $50 000)]**. At a TER of 6.0% and a DC of ASA of $10, the CER_min_ for preeclampsia is essentially the same as the TER with 0.05 or 0.52 QALYs gained (6.4% and 6.0%, respectively). At a TER of 6.0%, but a DC of ASA of $1000, the CER_min_ increases to 9.8% with 0.52 QALYs gained (dashed blue lines) and 46.0% with 0.05 QALYS gained (dashed red lines).

We can also determine how the CER_min_ changes with DC. Under the reality that ASA is cheap, we see that the TER very closely approximates the CER_min_ for even a small QALY gain (Fig. 4). If ASA were much more expensive, then our threshold to treat would increase dramatically, and few women could achieve the required level of risk.

### Application of MERT to preeclampsia prophylaxis with ASA

Sinclair et al. developed the *minimum event rate for treatment* (MERT) in order to arrive at the event rate (i.e., absolute risk) to justify starting a specific treatment for a given disease [21]. The equation for the MERT is as follows:

$$MERT=1/\left(NNP_{T}\times RRR\right)[21].$$

**Fig 5:**
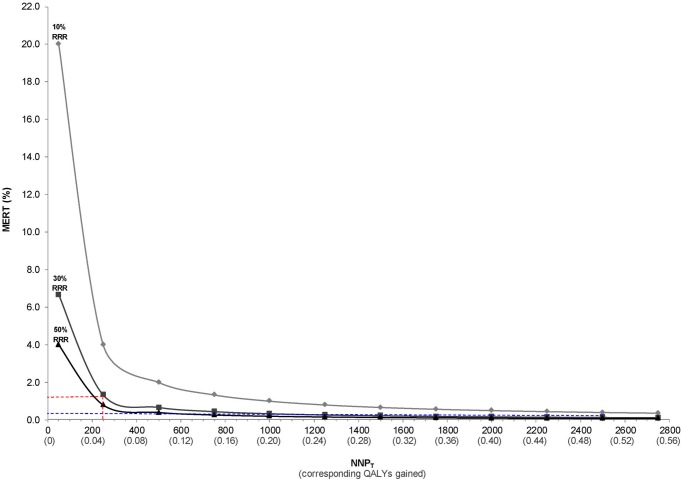
Minimum event rate for treatment (MERT) for ASA to prevent preeclampsia at varying thresholds of number needed to prevent (NNPT) and at 10%, 30% and 50% relative risk reductions (RRR) for the efficacy of ASA. NNP_T_ calculated as: (QALYs gained x $50000) / DC [24], assuming a direct cost (DC) of ASA of $10 per pregnancy. MERT is calculated as: 1 / (NNP_T_ x RRR) [21]. At a NNP_T_ of 250 (corresponding to 0.05 QALYs gained), the MERT is 1.3% (dashed red lines), which declines to 0.13% when the NNP_T_ is 2600 (corresponding to 0.52 QALYs gained, [dashed blue lines]).

As expected, the MERT changes with varying NNP_T_ [21], and is highest when the NNP_T_ (and corresponding QALY gained) is lowest (Fig. 5). Not only does the MERT decline (i.e., improve) with increasing NNP_T_, but so too does the MERT decline with greater efficacy of ASA, indicated by a higher RRR against developing PE (Fig. 5).

## Discussion

When initiated in the window between 12 to 16 weeks gestation, ASA effectively reduces the risk of PE and its related adverse perinatal outcomes, including intrauterine growth restriction, preterm birth, and neonatal death [11–14, 27]. To date, there is no objective estimate about the threshold of risk to warrant ASA prophylaxis in a given woman. This uncertainty may partly contribute to the variability in recommendations for ASA prophylaxis, or its uptake among practitioners, resulting in ASA being unnecessarily withheld in some women [16].

To help address this problem, we proposed using the CER_min_, which, based on the TER and NNP_T_, arrives at the absolute risk (i.e., threshold rate) at which prophylaxis is indicated. The CER_min_ declines as a preventive therapy becomes more effective and less costly. The CER_min_ is conceptually similar to the MERT [21], but the former relies on knowing the TER and NNP_T_, and the latter on knowing the RRR and NNP_T_. Both the CER_min_ and the MERT are estimated with a degree of uncertainty [28]. An advantage of the MERT is that RRRs conveniently express the magnitude of treatment effect and are transportable across populations with various baseline risk [29]. However, RRRs conceal the underlying absolute risks, while TERs do not.

One concern about using the equation for NNP_T_ by Shimbo et al. [24] is that it does not account for the costs saved by avoiding PE-related events, thereby overestimating the CER_min_ and the MERT. Additionally, it does not account for potential adverse effects (i.e., harm) conferred by a treatment and the resultant associated costs, in terms of DC and QALYs lost. However, in the specific case of ASA prophylaxis for PE, the NNP_T_ formula by Shimbo et al. appears valid, as ASA has no demonstrable adverse effects on the pregnancy, delivery or the neonate [3, 15]. As an example, one RCT assigning 9364 pregnant women either ASA or matching placebo found that ASA was not associated with significant increases in placental haemorrhages or in bleeding during preparation for epidural anaesthesia [30]. In the EAGeR trial, which assigned women with prior spontaneous pregnancy loss to ASA 81 mg daily (615 women) or placebo (613 women), from preconception until 36 weeks’ gestation or completion of 6 menstrual cycles, the risk of side effects and adverse events was the same in both groups [31]. However, in those ASA RCTs with smaller sample sizes, large beneficial effect sizes are more likely to be seen, while the chance of detecting rare adverse outcomes is reduced [32, 33].

The NNP_T_ values that we calculated for ASA prophylaxis were very high, and the subsequent CER_min_ and MERT values very low—a product of ASA’s extremely low cost and absence of known adverse effects. The MERT, even at conservative estimates of QALYs gained, was mostly under 1%, which is seemingly too low to truly reflect the absolute risk of PE that can justify ASA prophylaxis. The estimates of the CER_min_, though not as dramatically low as those for the MERT, were often barely higher than the TER. At such low CER_min_ or MERT values, a clinician might be tempted to recommend ASA to a woman with no known risk factors—ASA can conceivably prevent PE with no associated risks and a minimal cost. However, the clinician and/or patient may decide to forgo ASA prophylaxis when her risk of PE is low, and there remains the potential to introduce a rare side effect from ASA, and/or there is a minor inconvenience of taking ASA. A future study should measure the preferences of a group of caregivers and parents, to see if the risk threshold for starting ASA differs from the CER_min_ or MERT.

Although the methods used herein may slightly underestimate the threshold risk for PE to warrant ASA prophylaxis, our results suggest that eligible women need not be at “high risk” for PE, but rather, at some modestly elevated risk. In the development of a clinical prediction rule that identifies women at risk of PE, the threshold adopted to consider ASA prophylaxis may only need to be slightly higher than that of the general population, perhaps somewhere between 6% and 10% [11–14]. Ideally, the threshold range we have provided can help physicians choose the right woman in whom ASA prophylaxis is warranted. Nonetheless, using this threshold as a guide would be enhanced by having more information on the contributions of common clinical risk factors for the development of PE, independently, and in combination; that is, we need a very simple and accurate tool for determining a woman’s absolute risk of PE in the first trimester of pregnancy, before 12 to 16 weeks gestation. Finally, it is worthwhile to further investigate the efficacy and QALYs gained from ASA prophylaxis separately for the mother and her baby.

## Supporting Information

S1 Table Relative risk reductions for ASA versus control groups for preeclampsia and its associated short-term adverse obstetrical and perinatal outcomes. (DOCX)
