# Supplementary material for: Risk Threshold for Starting Low Dose Aspirin in Pregnancy to Prevent Preeclampsia: An Opportunity at a Low Cost
Source: PLoS One. 2015 Mar 19;10(3):e0116296. doi: 10.1371/journal.pone.0116296 (PMC4366221; doi:10.1371/journal.pone.0116296)
Supplement: S1 Table — (DOCX) [file pone.0116296.s001.docx]

# Table S1. Relative risk reductions for ASA versus control groups for preeclampsia and its associated short-term adverse obstetrical and perinatal outcomes.

|  |  | Relative risk reduction  (%, 95% confidence interval) [reference]  comparing ASA versus control | |
| --- | --- | --- | --- |
|  | Outcome | ASA started in first or second trimester of pregnancy | ASA started before 20 weeks gestation |
| *Preeclampsia* | Any preeclampsia | 10 (3 to 16) [[33](#_ENREF_33)] | 53 (35 to 66) [[14](#_ENREF_14)] |
|  | Severe preeclampsia | 35 (-7 to 60) [[13](#_ENREF_13)]74 (-26 to 95) [[14](#_ENREF_14)] | 82 (59 to 92) [[13](#_ENREF_13)] 78 (43 to 92) [[14](#_ENREF_14)] |
|  | Preterm-onset preeclampsia | Not available | 89 (67 to 96) [[12](#_ENREF_12)] |
| *Obstetric* | Caesarean delivery | -3 (-8 to 1) [[33](#_ENREF_33)] | -2 (-6 to 2) [[11](#_ENREF_11)] |
|  | Placental abruption | -13 (-48 to 13) [[33](#_ENREF_33)] | 38 (-403 to 92) [[14](#_ENREF_14)] |
| *Perinatal* | Preterm birth < 37 weeks | 7 (2 to 11) [[33](#_ENREF_33)] | 78 (51 to 90) [[14](#_ENREF_14)] |
|  | Intrauterine growth restriction | 20 (1 to 35) [[32](#_ENREF_32)] | 56 (35 to 70) [[14](#_ENREF_14)] |
|  | Neonatal mortality | 11 (-22 to 36) [[11](#_ENREF_11)] | Not available |
|  | Perinatal mortality | 11 (-8 to 26) [[11](#_ENREF_11)]13 (-10 to 31) [[13](#_ENREF_13)] | 18 (2 to 31) [[11](#_ENREF_11)]59 (8 to 81) [[13](#_ENREF_13)] |
